# Cross-architecture tuning of silicon and SiGe-based quantum devices using machine learning

**DOI:** 10.1038/s41598-024-67787-z

**Published:** 2024-07-27

**Authors:** B. Severin, D. T. Lennon, L. C. Camenzind, F. Vigneau, F. Fedele, D. Jirovec, A. Ballabio, D. Chrastina, G. Isella, M. de Kruijf, M. J. Carballido, S. Svab, A. V. Kuhlmann, S. Geyer, F. N. M. Froning, H. Moon, M. A. Osborne, D. Sejdinovic, G. Katsaros, D. M. Zumbühl, G. A. D. Briggs, N. Ares

**Affiliations:** 1 Department of Materials, University of Oxford, Parks Road, Oxford, OX1 3PH UK; 2 Department of Physics, University of Basel, Basel, 4056 Switzerland; 3 Institute of Science and Technology Austria, Am Campus 1, Klosterneuburg, 3400 Austria; 4 L-NESS, Dipartimento di Fisica, Politecnico di Milano, Polo di Como, ViaAnzani 42, Como, 22100 Italy; 5 Department of Engineering Science, University of Oxford, Parks Road, Oxford, OX1 3PJ UK; 6 Department of Statistics, University of Oxford, 24-29 St Giles, Oxford, OX1 3LB UK

**Keywords:** Physics, Applied physics, Condensed-matter physics, Quantum physics

## Abstract

The potential of Si and SiGe-based devices for the scaling of quantum circuits is tainted by device variability. Each device needs to be tuned to operation conditions and each device realisation requires a different tuning protocol. We demonstrate that it is possible to automate the tuning of a 4-gate Si FinFET, a 5-gate GeSi nanowire and a 7-gate Ge/SiGe heterostructure double quantum dot device from scratch with the same algorithm. We achieve tuning times of 30, 10, and 92 min, respectively. The algorithm also provides insight into the parameter space landscape for each of these devices, allowing for the characterization of the regions where double quantum dot regimes are found. These results show that overarching solutions for the tuning of quantum devices are enabled by machine learning.

## Introduction

Before we can use a quantum computer we first need to be able to turn it on. There are many stages to this initial step, particularly for quantum computing architectures based on semiconductors. Silicon and SiGe devices can encode promising spin qubits^1^, demonstrating excellent fidelities, long coherence times and a pathway to scalability^2–9^. Many of these key characteristics revolve around the material itself providing the opportunity to be purified to a near-perfect magnetically clean environment resulting in very weak to no hyperfine interactions. As the material of choice of the microelectronics industry, gate-defined quantum dots in silicon and SiGe have great potential for the fabrication of circuits consisting of a large number of qubits, an essential requirement to achieving a universal fault-tolerant quantum computer ^10,11^.

Multiple gate electrodes provide the ability to tune differing devices into similar operating regimes. These gate voltages define a large parameter space to be explored. Each device architecture and material realisation defines a specific parameter space. The time-consuming challenge of tuning semiconductor devices becomes intractable as we combine different device architectures in the realisation of complex quantum circuits with millions of components. In some cases it may take human experts over 3 h to tune a double quantum dot device^12^. The development of machine learning algorithms for quantum device tuning^12–27^ is exceptionally challenging when looking for such overarching solutions. Multiple algorithms address different parts of the tuning problem such as finding double quantum dots^13,22,25^, and then the optimisation^15,16,21,24^ and identification of transport features^18,19,26^. A significant number of tuning algorithms have been developed for AlGaAs/GaAs double quantum dots^13,14,16,17,23^. However, few have been demonstrated across different architectures nor on material compositions poised for scalability^20,28^, and only a small number of algorithms provide insight into the device parameter space^12^.

Here we demonstrate that it is possible to tune quantum dots in three different device architectures and material systems completely automatically. This machine learning-based algorithm, which we call ‘Cross-Architecture Tuning Solution using AI’ (CATSAI), requires only the following hyperparameters to be set once, for each type of device, in a configuration file: source-drain bias, safety voltage bounds, resolution and size of acquisition current maps and traces, the offset current noise floor, and Coulomb peak segmentation threshold (see Supplementary Material S1). The origin and gate voltage sweep directions can be arbitrarily selected for devices operating with accumulation or depletion mode gate electrodes, and either holes or electrons as majority charge carriers. An advanced signal processing classification method handles charge switches and other noise patterns.

We demonstrate our CATSAI algorithm for a Si accumulation-mode ambipolar FinFET^29–31^, a depletion-mode Ge/Si core/shell nanowire^32–34^ and a laterally-defined device in a Ge/SiGe heterostructure^35–38^, operating with holes as charge carriers. We show that CATSAI outperforms random search and human experts on all devices. Our machine learning-based approach also reveals the size and characteristics of the double quantum dot regime within the multidimensional parameter space defined by each gate voltage architecture. The demonstration of a general algorithm for the automatic tuning of a range of different devices with different noise profiles and compatible with industry manufacturing standards opens the path to building quantum circuits at scale for the next generation of quantum computers.

## Methods

**Figure 1:**
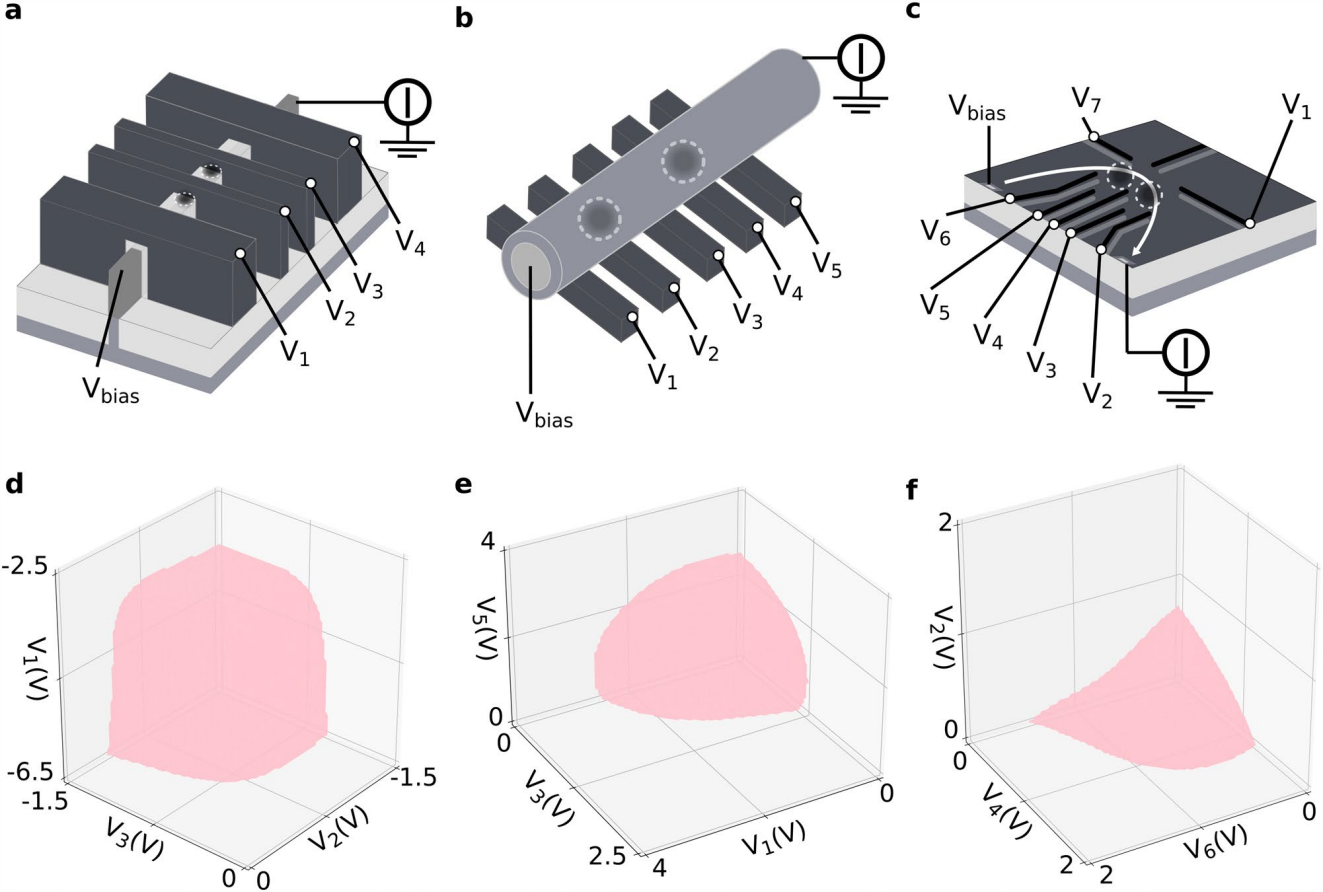
Device schematics. Si FinFET (**a**), GeSi nanowire (**b**) and Ge/SiGe heterostructure (**c**) device architectures and their corresponding current pinch-off hypersurfaces for hole transport calculated using a Gaussian process model for one of the tuning algorithm runs (**d**–**f**). Three gates are plotted for illustrative purposes with the remaining gates on each device set to a constant value. The bias was kept constant throughout the experiment. CATSAI was given control over the gate electrodes $V_{1}$–$V_{4}$, $V_{1}$–$V_{5}$, and $V_{1}$–$V_{7}$ on the FinFET, nanowire and heterostructure, respectively. The dashed white circles show the approximate locations of the quantum dots formed in the devices.

### The devices

Double quantum dots are defined by applying DC voltages to the gate electrodes $V_{1} - V_{4}$ for the FinFET, $V_{1} - V_{5}$ for the nanowire, $V_{1} - V_{7}$ for the heterostructure (Fig. 1). The dashed white circles show the approximate locations of the quantum dots formed in the devices. For the FinFET, the lead gate electrodes $V_{1}$ and $V_{4}$, open and close the quasi 1D silicon channel to charge carriers by controlling the size of the tunnel barrier between the quantum dots and the source and drain. The left and right plunger gate electrodes $V_{2}$ and $V_{3}$, control the occupation of the left and right quantum dot respectively. A current is driven through the FinFET by applying a bias voltage $V_{\textrm{bias}}$ of 7.6 mV (+ 3.8 mV at the source, - 3.8 mV at the drain) to NiSi contacts^29^. The gate voltages of the FinFET are operated such that the charge carriers are holes confined by accumulation. For the nanowire, gates $V_{2}$ and $V_{4}$ act as left and right plunger gates for the quantum dots formed within the 1D channel with the remaining gates mainly controlling the tunnel barriers. Hole quantum dots are formed in depletion mode. We set $V_{\textrm{bias}}=4$ mV. For the Ge/SiGe heterostructure, $V_{5}$ and $V_{3}$ operate as the left and right plunger gate electrodes respectively, with the remaining gate electrodes utilised as barrier gates. The white arrow denotes the flow of current. We set $V_{\textrm{bias}}=0.5$ mV and the charge carriers are holes confined in depletion mode. The values of $V_{\textrm{bias}}$ are set to be above typical charging energies for single quantum dots in each device. The choice of $V_{\textrm{bias}}$ can be left to an optimiser. For the heterostructure, experiments were performed at 300 mK, for the nanowire at 1.5 K and for the FinFET at 800 mK. The devices were operated at different temperatures based on access to different cryogenic equipment across laboratories.

Voltages applied to the gate electrodes of the devices can cause the current flow to pinch-off, transitioning from a relatively high current to a near-zero value. These voltages where pinch-off occurs define a hypersurface within the entire voltage space for each device. CATSAI has no knowledge of the device architecture and generates a model of the hypersurface after a given number of iterations. The resulting hypersurface for different devices is shown in Fig. 1d–f. Three gates are plotted for the ease of visualisation and the remaining gates are kept constant at their average value at pinch-off across the hypersurface (see Supplementary Material S1). The hypersufaces corresponding to different devices present different curvatures, leading to different tuning landscapes. The FinFET hypersurface (Fig. 1d) is near symmetrical in the plunger gates plane, $V_{2} - V_{3}$. This is expected as these gate electrodes are nominally identical. Although $V_{1}$ is wider than the plunger gates, its effect is not stronger. The curvature of the nanowire’s hypersurface is similar in the planes $V_{1} (V_{5}) - V_{3}$, since these planes are defined by the outer-middle barrier gates (Fig. 1e).

The heterostructure’s hypersurface has almost planar dependence on gate voltages $V_{2,4,6}$ (Fig. 1f). The hypersurface’s curvature in the $V_{2}$ – $V_{4}$ plane is evidently similar to that in the $V_{6}$ – $V_{4}$ plane, in agreement with the gate architecture. This hypersuface is qualitatively different to that reported for a relatively similar gate architecture patterned on a different heterostructure (AlGaAs/GaAs) ^12^. The more pronounced curvature of the hypersurfaces corresponding to the FinFET and the nanowire are expected given the larger gate couplings that are typically observed in FinFET and nanowire devices. Hypersurface characterisation could be used to inform device design and quantify device variability. Despite the stark differences in gate voltage landscapes, which evidence the difficulties of cross-architecture tuning, CATSAI is able to tune across all three device architectures.

**Figure 2:**
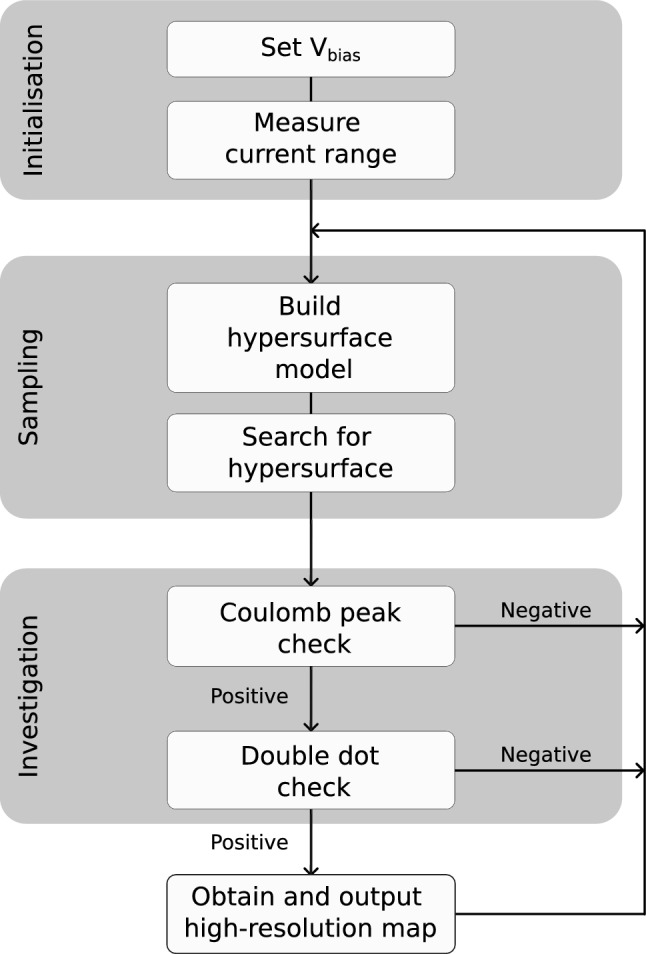
Outline of CATSAI’s workflow. The initialisation stage consists of setting $V_{\textrm{bias}}$ then measuring the maximum and minimum (offset) current flowing through the device. The sampling stage detects pinch-off locations in gate voltage space. The algorithm selects a vector in gate voltage space $\varvec{u}$ based on the model it generates of the hypersurface and of the probability of finding Coulomb peaks in a given location in gate voltage space. In the investigation stage the algorithm uses the plunger gates to sequentially acquire current traces and maps which are sent to the relevant classifiers. The Coulomb peak detector is a random forest classifier which determines whether Coulomb peaks are present (positive) or not (negative) within a current trace. In each iteration, the algorithm outputs a high-resolution current map if the double dot check score function is passed. After the investigation stage, the algorithm returns to the sampling stage.

### The CATSAI algorithm

CATSAI’s workflow consists of three stages, the initialisation stage, the sampling stage and the investigation stage (Fig. 2). In the initialisation stage $V_{\textrm{bias}}$ is fixed, and the current range, i.e. the maximum and minimum current flowing through the device, is determined by measuring the current both with all the gate electrodes set to 0 V and to their maximum permissible magnitude. To avoid damage to the device the algorithm is given voltage bounds in which it can operate each gate electrode. The bounds are known or determined in advance of running the algorithm.

After the initialisation stage, the algorithm turns to the sampling stage. The algorithm selects a vector $\varvec{u}$ in the gate voltage space of the device based on a Gaussian process model of the hypersurface as shown in Fig. 1d–f, and a weighting from the probability of finding Coulomb peaks at a given location in voltage space ${\tilde{\textrm{P}}_{peaks}}$ (see Supplementary Material S1). This vector consists of all the gate voltages considered for tuning. The algorithm then sweeps the gate voltages along that direction until pinch-off occurs. The algorithm identifies the onset of pinch-off as a current drop below a certain threshold ($0.5\%$ of the measured current range). The *N*-dimensional hypersurface is delimited by the pinch-off voltages of the *N* gate electrodes for each device.

At the start of the investigation stage, once pinch-off is found in a given gate voltage direction, a high-resolution current trace is performed. This current trace, which starts at the pinch-off location and runs diagonal within the plane defined by the plunger gates, was set to have a fixed length of 128 pixels and resolution 1.56 mV/pixel for the nanowire and 0.78 mV/pixel for the FinFET and the heterostructure. The plunger gates, selected before running the algorithm, are those expected to predominantly shift the electrochemical potential in left and right dots. Using a random forest classifier^39,40^, the algorithm determines whether Coulomb peaks are present in the current trace. Due to prior training this approach is robust against noise and switches unlike simple peak-finding packages which are much more likely to be tricked that a trace of noise corresponds to hole/electron transport as they typically rely on the sole identification of local maxima. This classifier is key to the success of CATSAI across device types with different noise characteristics (see Supplementary Material S1).

If Coulomb peaks are found by the classifier then a low-resolution current map ($16 \times 16$ pixels, 5 mV/pixel for the nanowire and 9 mV/pixel for the FinFET and the heterostructure) is taken by sweeping the plunger gates. The current map is believed to contain double quantum dot features if it scores above a threshold, which is fixed and can be optimised. We use the same score function as in Ref.^12^. If double quantum dot features are believed to be present, a high-resolution current map ($48 \times 48$ pixels, 4.2 mV/pixel for the nanowire and 2.5 mV/pixel for the FinFET and the heterostructure) is taken. At the end of the iteration, CATSAI returns to the start of the sampling stage. CATSAI proceeds to update the hypersurface model and ${\tilde{\textrm{P}}_{peaks}}$ with the knowledge garnered of pinch-off and Coulomb peak locations respectively. CATSAI runs for a certain number of iterations. A posteriori, to gauge the algorithm’s performance, humans can verify if the double quantum dot features were successfully identified by the algorithm.

CATSAI is benchmarked against a version of this algorithm which does not use a weighted hypersurface model to influence the sampling of the hypersurface. It instead samples a point in the voltage parameter space of the device at random and carries out the investigation stage for each iteration. We call this version of CATSAI ‘Random Search’, although it is important to highlight that it still relies on peak detection. In this manner we are able to gain insight into the online performance of our Coulomb peak detector. Moreover, it enables cross-validation of CATSAI’s sampling method which could not be achieved otherwise, given the lack of alternative algorithms suitable for this tuning task.

### Tuning across architectures and material systems

**Figure 3:**
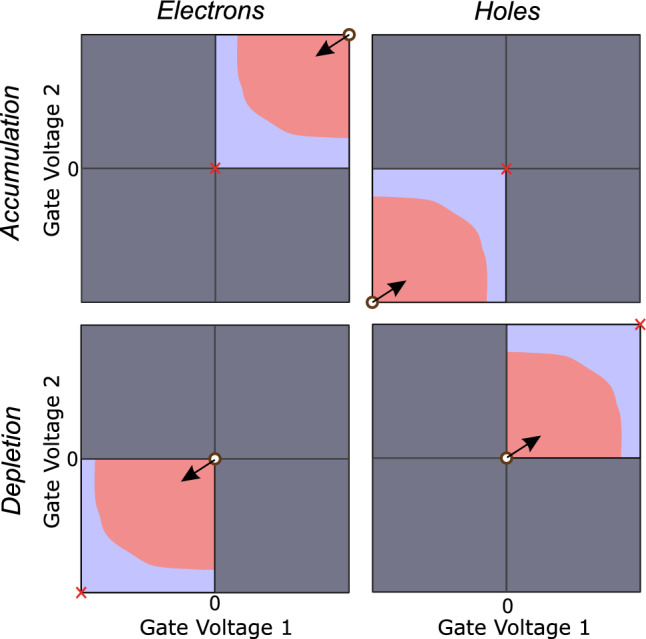
Gate-voltage space exploration. Different charge carriers (gate operation modes) are represented in different columns (rows). Each panel illustrates the initial placement of the origin (white circle), search boundary (red cross), and search direction (black arrow). The gate voltage space is divided into regions of near-zero (blue) and non-zero (pink) current. Regions of voltage space which cannot be explored due to the gate voltage bounds set to avoid device damage are greyed out.

To make the algorithm general across different charge carriers and modes in which gate electrodes are designed to act (depletion or accumulation), the origin, bound, and direction of the gate-voltage space exploration used in the sampling stage are set in a configuration file (Fig. 3). The algorithm starts in the gate voltage configuration which delivers the highest current and sweeps gate voltages in the direction of decreasing current with the aim of locating the boundary between the two regions. This flexibility in the search of gate voltage space, combined with a noise-tolerant classification of Coulomb peaks in the investigation stage, makes CATSAI robust across device architectures and material systems. The Coulomb peak classifier is trained on current traces acquired in different Si FinFET and GeSi nanowire devices (see Supplementary Material S1). This random forest classifier can successfully handle both noise and charge switches, resulting in a robust Coulomb peak detection. The number of false positives in the classification that are accepted for the next step of the investigation stage is thus reduced, significantly shortening device tuning times.

## Results

**Figure 4:**
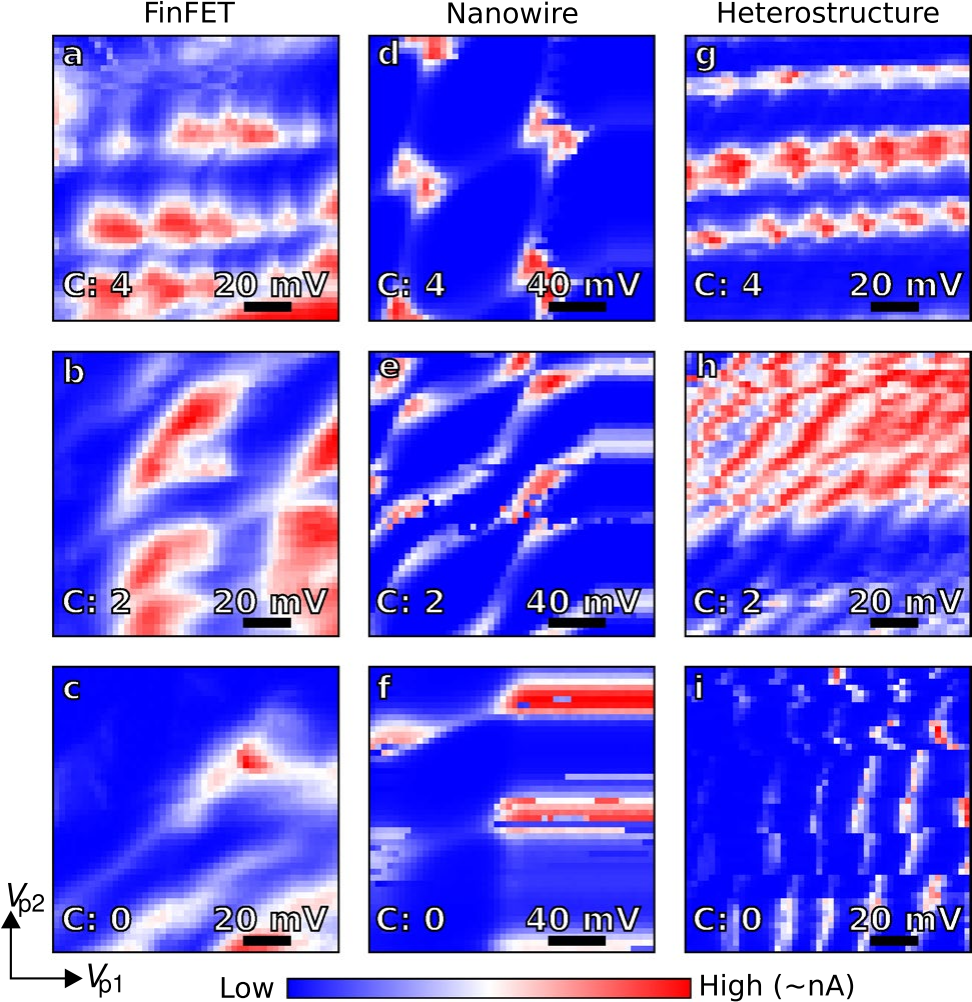
Device tuning. Examples of current map outputs on the different devices in which CATSAI was run. High resolution maps are generated during the investigation stage by sweeping the plunger gates of each device $V_{p1,p2}$; for the FinFET $V_{3,2}$ (**a**–**c**), the nanowire $V_{4,2}$ (**d**–**f**) and the heterostructure $V_{3,5}$ (**g**–**i**). These current maps are labelled *a posteriori* by humans to verify whether they correspond to the double quantum dot regime. *C* indicates the number of humans out of four who labelled the current map as corresponding to a double quantum dot regime. Red (blue) indicates regions of high (low) current in each map.

**Figure 5:**
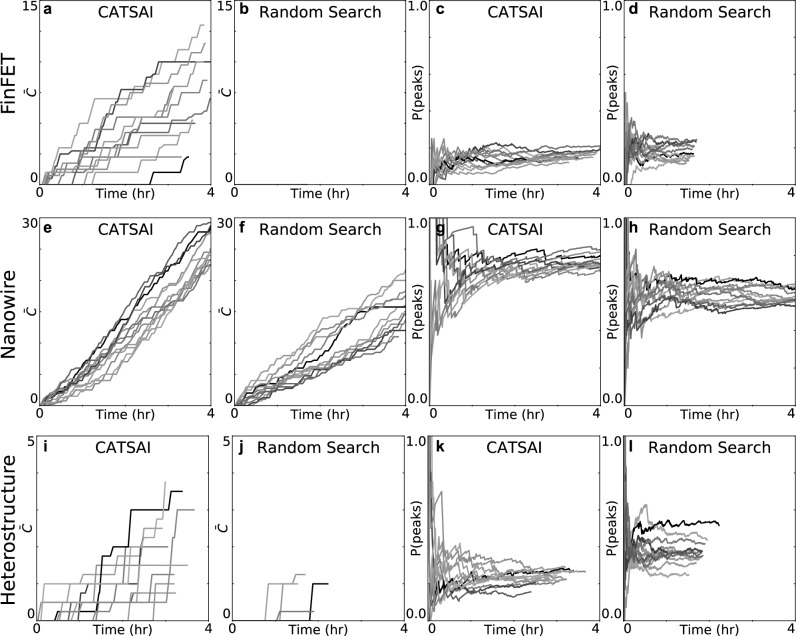
Benchmarking the algorithm’s performance. Cumulative sum of the average number of double quantum dot regimes verified by humans $\bar{C}$ (first and second columns) and probability of finding Coulomb peaks $\mathrm {P(peaks)}$ (third and fourth columns), as a function of laboratory time for each run of CATSAI and Random Search algorithms. Each coloured line corresponds to a different run. Rows correspond to the different devices. Only the first 4 h of each tuning run are shown for ease of visualisation. CATSAI outperforms Random Search in the number of double quantum dot regimes located for all devices. The value of $\bar{C}$ remains at 0 in many of the Random Search runs, and thus are not visible in the plots of $\bar{C}$ as a function of time. The increase in $\mathrm {P(peaks)}$ as a function of laboratory time observed for the CATSAI runs after the first 12 iterations can be explained by the algorithm ‘learning’ a better model of the hypersurface as the Gaussian process regression acquires more observations.

The algorithm was run for 250 iterations for all experiments performed. The number of iterations that the algorithm runs without a hypersurface model, i, which can be separately optimised, was fixed to twelve in this case. A few examples of output current maps identified by CATSAI as containing double quantum dot features for the different devices considered are displayed in Fig. 4. The double quantum dot regimes pictured in Fig. 4 show that our Algorithm is capable of identifying double dot regimes despite device characteristics that might be far from ideal. Although accurate most of the time, the score function that the algorithm uses to detect double quantum dot regimes can sometimes be tricked by charge switches, as observed in Fig. 4i. The ideal double dot regimes found by the algorithm are tuned to a point where a specialised fine tuning algorithm such as that developed by van Esbroeck et al.^24^ would be suitable to follow on and optimise transport features.

To benchmark the performance of the algorithm, the output current maps were labelled by human experts at the end of the tuning experiment to verify whether they corresponded to the double quantum dot regime (see Supplementary Material S1). The human experts were unaware whether the current maps to be labelled were the output of CATSAI or Random Search. We asked multiple human experts to label the data as each human expert may have different criteria as to what constitutes a double quantum dot or not for example, well-defined bias triangles or a rough honeycomb pattern may suffice. We define *C* as the number of humans who labelled a current map as containing double quantum dot features. In each iteration of the algorithm, we cumulatively sum the value of *C* normalised by the total number of human labellers (four). The resulting quantity, $\bar{C}$, provides a measure of the number of double dot regimes found by the tuning algorithm while considering disagreements between human labellers.

Figure 5a–j shows $\bar{C}$ as a function of laboratory time for 12 runs, where each line in Fig. 5a–j corresponds to a different run, of CATSAI and Random Search for each of the devices considered. CATSAI outperforms Random Search in the total number of double quantum dot regimes located in all cases. The Random Search algorithm did relatively well in locating double quantum dot regimes in the nanowire but did not locate any double quantum dot regime in the FinFET (Fig. 5b) and struggled to locate more than one double quantum dot regime in the Ge/SiGe heterostructure device (Fig. 5j).

The probability of Coulomb peaks estimated for a given number of iterations, $\mathrm {P(peaks)}$, is plotted as a function of laboratory time for each algorithm run and each device in Fig. 5c–l. For the Random Search and the first i iterations of CATSAI, the algorithm chooses pinch-off locations randomly, and thus $\mathrm {P(peaks)}$ does not show a definite trend. For the subsequent iterations, we expect CATSAI to learn which are the promising locations in gate voltage space, and $\mathrm {P(peaks)}$ should thus increase as a function of time. This increase would not be monotonic, since the algorithm balances an exploration/exploitation trade-off ^12^.

The trend of $\mathrm {P(peaks)}$ as a function of laboratory time observed in most CATSAI runs is similar for the FinFET, nanowire and the heterostructure devices. $\mathrm {P(peaks)}$ has a gradual upward trend in many of the experimental runs after the first 30 min to an hour and then saturates over laboratory time at different values between the devices. The saturation after 1–2 h is expected given that transport feature can only be found in a limited portion of the gate voltage space.

For the FinFET device and the heterostructure, the values of $\mathrm {P(peaks)}$ are on average larger for Random Search than for CATSAI runs. However, the number of double dots found by Random Search is still less than CATSAI in both devices. The value of $\mathrm {P(peaks)}$ from the Random Search runs in these devices is inflated due to false positive classifications by the Coulomb peak classifier, confirmed by human labels of all the current traces (see Supplementary Material S1).

**Table 1:** Median device tuning times with 80% credible intervals (equal tailed) corresponding to CATSAI and Random Search algorithm runs for all devices considered.

Device	Tuning times (min)	
	CATSAI	Random search
GeSi nanowire	9.5 (6.7, 12)	17 (9.9, 26)
Si FinFET	30 (26, 37)	–
Ge/SiGe Het.	92 (71, 120)	360 (190, 830)

The random search tuning time for the FinFET is unknown as no double quantum dot regimes were located.

CATSAI tuned all devices faster than Random Search. The median tuning times are 10 min for the nanowire, 30 min for the FinFET, and 90 min for the heterostructure (Table 1). The Random Search algorithm was surprisingly quick at tuning the nanowire, while unable to tune the FinFET successfully within 12 runs of the algorithm, which totals a laboratory time of 19 h. We estimate credible intervals as described in Ref.^12^. Reduced tuning times for the FinFET device could probably be achieved by fixing or grouping the lead gate voltages. The difference between the upper and lower credible interval of the tuning times achieved in the heterostructure device is an order of magnitude less than that achieved by Random Search.

The difference between median tuning times for different devices begs the question whether the dimensionality of the gate voltage space is the key factor affecting tuning times or if there is a more subtle characteristic at play. The faster median tuning times were achieved in those devices for which the gate voltage space has fewer dimensions, i.e. the FinFET and the nanowire. Although the nanowire does have greater gate electrode dimensionality than the FinFET, we still observe faster tuning times for the nanowire. There would seem to be more double quantum dot regimes in the nanowire gate voltage space than there are in that of the FinFET.

This hypothesis is reinforced by the lack of double quantum dot regimes found in the FinFET by Random Search and it is in agreement with the experience of human experts when tuning these devices.

A reason for the lack of double quantum dot regimes is the sharp pinch-off that occurs as a function of the lead gate electrodes. The probability of finding lead gate voltages that enable current flow and plunger gate voltages that lead to double quantum dot regimes is inherently low. As mentioned previously, faster tuning times for FinFETs would thus be expected for CATSAI and Random Search if the lead gate voltages, $V_{1}$ and $V_{4}$, are fixed.

**Figure 6:**
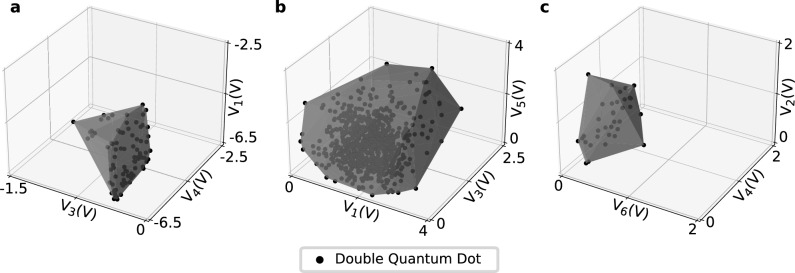
Double quantum dot regime volumes. Regions of voltage space (grey) encapsulate and define a volume where double quantum dots were found (black points) across all experimental runs of both random search and CATSAI in the FinFET (**a**), nanowire (**b**), and heterostructure (**c**). Three gates are plotted for illustrative purposes.

The parameter space in which double quantum dots were found, by labeller majority vote, across all experimental runs varies greatly between devices (Fig. 6). In the 3D-parameter space shown in Fig. 6 the double quantum dot regime volumes are 1.21 $\mathrm {V^3}$, 17.7 $\mathrm {V^3}$, 0.215 $\mathrm {V^3}$ for the FinFET, nanowire and heterostructure respectively. The FinFET (Fig. 6a) displays an almost planar voltage space across $V_{3}$, $V_{1}$ and $V_{4}$ which encapsulates the double quantum dots found. $V_{1}$ and $V_{4}$ display their symmetrical weighting by forming the base of the plane which is almost square, and centred at similar points in voltage space with an approximate area of 2 V $\times$ 2 V. This supports the thesis that tuning times could be reduced by grouping the lead gate electrode voltages. The nanowire (Fig. 6b) displays a much larger region in voltage space where double quantum dots were found across $V_{1}$, $V_{3}$ and $V_{5}$. In the parameter space pictured, there are areas of the double quantum dot regime volume which are more dense with double quantum dots than others. At the upper bounds of $V_{3}$ and $V_{5}$, double dots are present but sparse in the parameter space. However, the relatively high number of double dots across a wide parameter space provides evidence as to why the nanowire has the shortest tuning time and confirms that it has the most double dot regimes. The heterostructure displays the smallest volume across the parameter space of $V_{2}$, $V_{4}$ and $V_{6}$ and the double quantum dots are distributed more sparsely within the volume when compared to the two other devices. The combination of these two factors supports why the tuning times were the longest for the heterostructure in addition to its higher gate electrode dimensionality.

Beyond the 3D volumes displayed in Fig. 6, our Algorithm explores the full dimensionality of the available parameter space (4, 5, and 7 dimensional for the FinFET, nanowire and heterostructure, respectively), allowing us to gain new insights into the variability of operating regimes. The double quantum dot regime convex hull volumes, defined by the shape of the smallest convex set which encapsulates all double quantum dots found in the entire parameter space, are ${16.6\,\times \, 10^{-2}}\, \mathrm {V^4}$, 23.8 $\mathrm {V^5}$, ${26.3\,\times \, 10^{-4}}\, \mathrm {V^7}$ for the FinFET, nanowire and heterostructure, respectively. We name this metric, the Double Dot Voltage Space Volume (DDV). To put the DDVs into perspective we can scale them by the size of the parameter space of each device defined by the gate voltage bounds. The DDVs of the FinFET, nanowire and heterostructure occupy 0.174%, 5.95%, and 0.00206% of the device parameter spaces (95.1 $\mathrm {V^4}$, 400 $\mathrm {V^5}$, and 128 $\mathrm {V^7}$) respectively. Comparisons between these values must take into consideration the effect of gate voltages on the confinement potential for each device architecture, the presence of disorder, strain, and material characteristics. Still, this percentage gives us an insight into the ease of tuning each device architecture. Our Algorithm’s proficiency in effectively exploring diverse DDVs across various device architectures showcases its versatility and robust capabilities. The DDV metric can be used to explore differing quantum device architectures and materials, and thus has a wide-ranging of applicability.

## Discussion

We demonstrated fully automated tuning of quantum devices across material compositions and gate architectures. We achieved fast tuning times in a Si FinFET, a GeSi nanowire and a Ge/SiGe heterostructure device, three different types of devices with very different characteristics. The tuning times reported are as low as 30, 10 and 92 min respectively. Although there exist bespoke algorithms for tuning double quantum dots^20,21,41^, our algorithm is one of the few algorithms^28^ to demonstrate its capabilities and characteristic generality of tuning across different architectures and material systems. Moreover, our algorithm provides insight into the parameter space across the different types of devices tuned. The capability to tune these devices from scratch completely automatically, prepares the pathway laid out for the scaling of semiconductor qubits that lend themselves to industrial scale manufacture.

An analysis of the hypersurfaces corresponding to different device types and material systems could minimise variability and boost device performance by an informed device design. The size of the gate voltage space is also an important consideration in this context. While the FinFET and the nanowire gate-voltage spaces at mV resolution have approximately $10^{14}$ and $10^{17}$ pixels respectively, the mean tuning times are only different by a factor of 3, and surprisingly the median tuning time is shorter for the nanowire device.

The heterostructure, with a gate voltage space at mV resolution of $10^{23}$ pixels, shows a mean tuning time only 3 times longer than the nanowire. This would suggest that other factors, such as the design of the gate architecture and the disorder potential, might have a very significant role in how quickly a device can be tuned. Faster tuning times could be achieved by using device information, for example by grouping gate electrodes with similar functions. While the size of the gate voltage space is determined both by device properties and fabrication methods, the volume of the hypersurface and the volume of gate voltage space in which transport features are found could be useful to quantify device variability and to characterise and design different device architectures. This includes calculating how the hypersurface of a particular architecture is different between devices or thermal cycles via point set registration as well as Coulomb peak occurrence and Coulomb peak sensitivity within parts of the voltage space^42^. Additionally, our introduction of the new metric, Double Dot Voltage Space Volume (DDV), opens the door to understanding the sensitivity of the quantum dots’ confinement potential to each gate voltage value, and to examine the role of each gate electrode as plunger, barrier gates, etc. between devices. The DDV metric can be used to explore differing quantum device architectures and materials, and thus has a wide range of applicability.

We expect our Algorithm to be successful in tuning geometries where gate electrode cross-talk is more considerable. Moreover, our machine learning-based approach is geared towards navigating intricate parameter spaces rather than relying on a procedural algorithm workflow. As devices scale leading to more complex architectures and the number of quantum dots to tune grows, one could envisage tuning quantum dot arrays by using our Algorithm to tune a series of double quantum dots sequentially while deploying (novel) charge state compensation methods^43^.

Radio-frequency reflectometry measurements would also lead to faster tuning times and the possibility of efficiently tuning large device arrays. Our work evidences the potential of machine learning-based algorithms to find overarching solutions for the control of complex quantum dot systems.

### Supplementary Information

Supplementary Information.

## Data Availability

The data acquired by the algorithm during experiments is available from the corresponding author upon reasonable request.
