# Supplementary material for: Cross-architecture tuning of silicon and SiGe-based quantum devices using machine learning
Source: Sci Rep. 2024 Jul 27;14:17281. doi: 10.1038/s41598-024-67787-z (PMC11283483; doi:10.1038/s41598-024-67787-z)
Supplement: Supplementary file 1 — Supplementary Information. [file 41598_2024_67787_MOESM1_ESM.pdf]

Cross-architecture Tuning of Silicon and SiGe-based Quantum Devices Using Machine Learning

B. Severin,[1] D. T. Lennon,[1] L. C. Camenzind,[2] F. Vigneau,[1] F. Fedele,[1] D. Jirovec,[3] A. Ballabio,[4] D. Chrastina,[4] G. Isella,[4] M. de Kruijf,[2] M. J. Carballido,[2] S. Svab,[2] A. V. Kuhlmann,[2] S. Geyer,[2] F. N. M. Froning,[2] H. Moon,[1] M. A. Osborne,[5] D. Sejdinovic,[6] G. Katsaros,[3] D. M. Zumbühl,[2] G. A. D. Briggs,[1] and N. Ares[5]

[1]*Department of Materials, University of Oxford, Parks Road, Oxford, OX1 3PH, UK*
[2]*Department of Physics, University of Basel, Basel, 4056, Switzerland*
[3]*Institute of Science and Technology Austria, Am Campus 1, 3400 Klosterneuburg, Austria*
[4]*L-NESS, Dipartimento di Fisica, Politecnico di Milano, Polo di Como, ViaAnzani 42, 22100 Como, Italy*
[5]*Department of Engineering Science, University of Oxford, Parks Road, Oxford OX1 3PJ, United Kingdom*
[6]*Department of Statistics, University of Oxford, 24-29 St Giles, Oxford, OX1 3LB, UK*

(Dated: June 30, 2024)

SUPPLEMENTARY MATERIAL

Supplementary Methods

Devices

The FinFET [1], possesses a fin height of ∼ 15 nm, and a width of ∼ 20 nm and is defined on a near-intrinsic silicon substrate by means of electron beam lithography and dry etching. The gate stack consists of a ∼ 10 nm thick SiO_2 layer, covered by a ∼ 40 nm thick layer of TiN to form the gate electrodes. The gate widths are ∼ 15 nm, with a gate-to-gate separation of ∼ 35 nm, except the lead gates (V_1 and V_4) which have a width of ∼ 500 nm. The source-drain contacts are NiSi Schottky barriers. The nanowire [2], has a Ge core diameter of ∼ 20 nm and a ∼ 2 nm thick Si shell. The five Ti/Pd (1 /12 nm) gate electrodes are lithographically defined on p++-doped Si substrate covered with 290 nm thermal oxide. The gates have a width of 20 nm and a pitch of 50 nm. The gates are covered with a 20 nm thick layer of HfO_2. Electrical contact to the nanowire is made via Ti/Pd (0.5 / 60 nm) ohmic source-drain contacts. The Ge/SiGe [3] heterostructure ($Si_{0.3}Ge_{0.7}$/Ge/$Si_{0.3}Ge_{0.7}$) has a Ge quantum well thickness of ∼ 20 nm and it is buried below a ∼ 20 nm thick SiGe spacer. The oxide is a 20 nm thick layer of Al_2O_3 and above which reside Ti/Pd (3 /27 nm) gates each with a width of ∼ 30 nm. The ohmic contacts are Pt.

3D hypersurface plots

The 3D plot of the hypersurface for each device was generated by relying on the same method that CATSAI uses to generate the hypersurface of each device as it proceeds to coarsely tune it. The main difference being that no sampling is involved; the surface is generated by a model that makes use of the pinch-off locations detected during an algorithm run selected at random (CATSAI run 10). The model of the hypersurface used was a Gaussian Process (Matern52 Kernel). This model is then used as an interpolation method to generate the 3D plots; regularly spaced points in gate voltage space are considered and the model is used to determine whether these points lie within the hypersurface. The gate electrodes not considered for the plots are kept constant at their respective mean gate voltage values for which pinch-off was observed during the experiment (Supplementary Table III).

CATSAI's Workflow

For the first i (12) iterations of the sampling stage (Supplementary Fig. 1), the algorithm selects a vector $\boldsymbol{u}$ at random in the gate voltage space of the device since the algortithm is unaware of the characteristics of the device. This vector consists of all the gate voltages considered for tuning. The algorithm then sweeps the gate voltages along that direction until pinch-off occurs.

After the ith iteration, a model of the hypersurface is built using a Gaussian process and $\boldsymbol{u}$ is chosen by incorporating the knowledge gained during the peak detection module in the investigation stage. The algorithm achieves this by generating a set of candidate pinch-off locations on the hypersurface and using the probability of finding Coulomb peaks in a given location of gate voltage space, $\tilde{\mathrm{P}}_{\mathrm{peaks}}$, as a weighting for the choice of $\boldsymbol{u}$ [4]. Using Thompson sampling, the algorithm then selects one of the candidate pinch-off locations, defining a new $\mathbf{u}$. In each of the following iterations, the pinch-off locations and the information gathered by the peak detection are used to update the hypersurface model and $\tilde{\mathrm{P}}_{\mathrm{peaks}}$, respectively.

For the low resolution current map score function in the investigation stage, the algorithm is given the noise floor and the current at which Coulomb peaks can be segmented, a current value between the noise floor and the peak of the Coulomb peaks, the segmentation threshold. For simplicity, measurements of the safe bounds, noise floor and segmentation threshold are taken manually before running CATSAI; these measurements can easily be

automated.

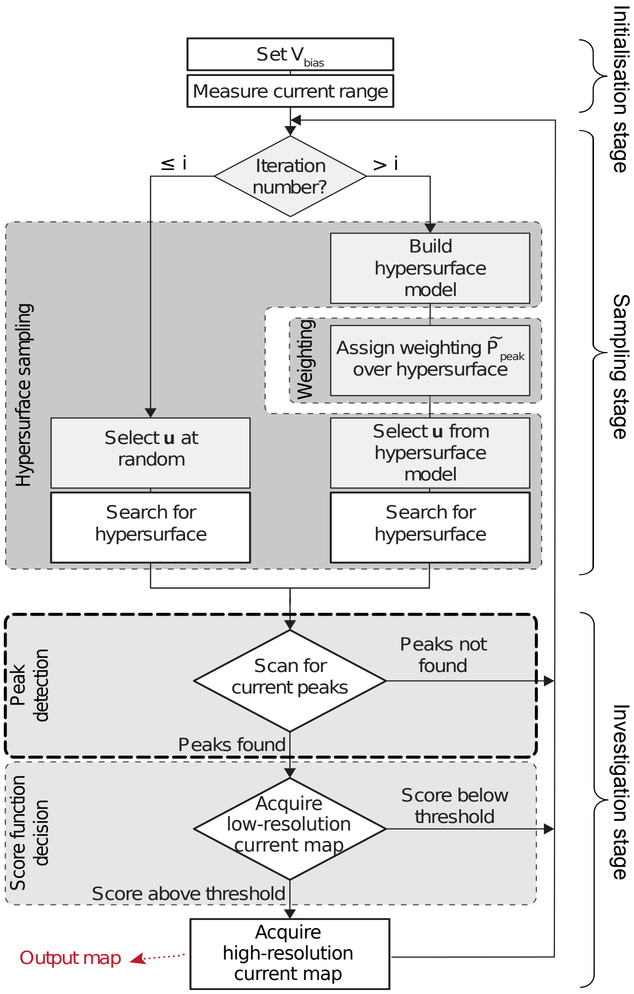

Supplementary Figure 1. CATSAI's workflow. For the first i iterations (left-hand branch of the sampling stage), the algorithm selects $\boldsymbol{u}$ at random and travels along it until the hypersurface is found. After the ith iteration (right-hand branch of the sampling stage), the algorithm selects $\boldsymbol{u}$ based on the model it generates of the hypersurface and of the probability of finding Coulomb peaks in a given location in gate voltage space, $\tilde{P}_{peaks}$. In the investigation stage the algorithm sweeps the plunger gates to generate current traces and low-resolution and high-resolution current maps if the conditions are satisfied for each classifier. Figure adapted from [4].

Coulomb peak detector

Due to the different types of current noise observed for each of the devices considered, a robust Coulomb peak detector was required. We thus developed a random forest Coulomb peak classifier.

A set of 128-pixel current traces was obtained running the tuning algorithm developed by Moon et al. [4] on different devices to those for which CATSAI was tested (Supplementary Table I); two different 5-gate GeSi nanowires (400 mV-long current traces), and a single 3-gate Si FinFET (200 mV-long current traces). We gathered 1095 current traces from GeSi nanowire device 1, 1321 from GeSi nanowire device 2, and 4306 from the Si FinFET device 1. The 6722 current traces were labelled by a single labeller (Brandon Severin), from which there were 553 labelled as positive (current traces containing Coulomb peaks) and the remainder (6169 current traces) were labelled as negative. 553 negative examples were randomly picked from the shuffled 6169 negative examples, to make up an even dataset of 1106 current traces. The breakdown of the data subsets include, for the positives: 115 traces from GeSi nanowire device 1, 100 from GeSi nanowire device 2 and 338 from the Si FinFET device 1. For the negative subset: 83 from GeSi nanowire device 1, 113 from GeSi nanowire device 2, and 357 from the Si FinFET device 1. Randomly chosen current traces from the even dataset of 1106 current traces were used to train and test the random forest Coulomb peak classifier; 70% of the traces chosen were used to train the classifier, and 30% were used to test it. No characteristic feature engineering or data pre-processing was done other than normalisation. The characteristic features the random forest classifier was trained on were the normalised current values of each trace at each pixel point, thus each sample had 128 characteristic features. The classifier relies on the Scikit-learn's ensemble RandomForestClassifier package [5]. An accuracy of 84% was achieved. The random forest classifier was then retested on 1562 current traces from a 5-gate Ge/SiGe heterostructure device 1 and an accuracy of 92% was achieved (Supplementary Table I, Test 2). This relatively high accuracy contrasts the Coulomb peak detector used in Ref. [4], which achieved an accuracy of 20% classifying the current traces obtained for the Ge/SiGe heterostructure device 1.

Coulomb peak detector: online performance

All the current traces after the CATSAI and Random Search experimental runs were complete were labelled by Brandon Severin. The actual human labels were compared against the labels predicted by the random forest Coulomb peak classifier used in the experiment (Supplementary Table II). The accuracy of the Coulomb peak detector is as follows, FinFET: 82.1%, 71.3%, nanowire: 86.0%, 82.3%, and heterostructure: 63.7%, 79.7% for all the Random Search and CATSAI runs respectively.

Algorithm configuration for the different type of devices studied

Across all devices the initialisation of the algorithm is set to 12 iterations (the first 5% of the total number of iterations for each run). In this work we did not apply any

pruning rules [4]. When searching for the hypersurface, the algorithm looks for current drops below 0.5% of the maximum current range. The parameters chosen to run the algorithm can be separately optimised. The model of the hypersurface is built via a Gaussian Process as in Ref. [4].

Other configuration parameters depend on the type of device to be explored (Supplementary Table IV & V). These parameters include: voltage bounds (origin and limit) set for each gate electrode to prevent device damage, the value at which the bias voltage is fixed, the noise and segmentation thresholds, and the size in gate voltage space of current traces (diag_trace), as well as low and high resolution current maps (2d_lowres and 2d_highres, respectively). During the investigation stage the current traces have a length of 128 pixels, the low resolution current maps have a size of 16 × 16 pixels, and the high resolution have a size of 48 × 48 pixels. The dimensions of the traces and the scans in voltage space are device dependent (Supplementary Table V).

The bias voltages were chosen to be slightly larger than typical charging energies expected for single quantum dots in each device. The noise and segmentation thresholds were chosen according to expected values; these can easily be replaced by a fixed percentage of the maximum-minimum current range across devices. The size of current traces and current maps in the investigation stage was larger for the GeSi nanowires, since the gate lever arms in these devices is often smaller compared to the other devices. These hyperparameters could also be optimised in future implementations. All hyperparameters were determined in advance before running the algorithm.

Labelling procedure

The current maps that are classified by the Algorithm as corresponding to a double dot regime are checked and labelled by human beings at the end of the experiment to benchmark the Algorithm's performance (Supplementary Table VI & VII). There is often disagreement between humans about what current maps correspond to a double quantum dot regime. The current maps for each type of device were thus labelled by 4 different and independent human labellers. Three datasets were collected, one for each device (nanowire, heterostructure and FinFET). For each device, the current maps collected by Random Search and CATSAI were grouped together and shuffled to avoid labellers' confirmation bias. Median tuning times were calculated using a Bayesian model based on the resultant labels as in Ref. [4].

Supplementary References

[1] Kuhlmann, A. V., Deshpande, V., Camenzind, L. C., Zumbühl, D. M. & Fuhrer, A. Ambipolar quantum dots in undoped silicon fin field-effect transistors. *Applied Physics Letters* **113**, 122107 (2018).

[2] Froning, F. N. *et al.* Single, double, and triple quantum dots in Ge/Si nanowires. *Applied Physics Letters* **113**, 73102 (2018).

[3] Jirovec, D. *et al.* A singlet-triplet hole spin qubit in planar ge. *Nature Materials* **20**, 1106–1112 (2021).

[4] Moon, H. *et al.* Machine learning enables completely automatic tuning of a quantum device faster than human experts. *Nature Communications* **11**, 4161 (2020).

[5] Pedregosa, F. *et al.* Scikit-learn: Machine learning in Python. *Journal of Machine Learning Research* **12**, 2825–2830 (2011).

Supplementary Tables

Device	Train	Test 1	Test 2	Algorithm run
GeSi Nanowire 0	-	-	-	x
GeSi Nanowire 1	x	x	-	-
GeSi Nanowire 2	x	x	-	-
Si FinFET 0	-	-	-	x
Si FinFET 1	x	x	-	-
Ge/SiGe Heterostructure 0	-	-	-	x
Ge/SiGe Heterostructure 1	-	-	x	-

Supplementary Table I. Devices used throughout this work. All devices used for training and or testing are different to the devices used in the experiment. Devices used for the experiment algorithm runs only are numbered as zero.

Supplementary Table II. Confusion matrices of the Coulomb peak detector for all of the experimental runs comparing actual human labels against the predicted labels of the random forest Coulomb Peak detector.

	Ge/SiGe Het. (Random Search)			Si FinFET (Random Search)			GeSi Nanowire (Random Search)	
	Pred. Neg.	Pred. Pos.		Pred. Neg.	Pred. Pos.		Pred. Neg.	Pred. Pos.
Act. Neg.	1878	1079	Act. Neg.	2455	512	Act. Neg.	1114	277
Act. Pos.	10	33	Act. Pos.	24	9	Act. Pos.	144	1465
	Ge/SiGe Het. (CATSAI)			Si FinFET (CATSAI)			GeSi Nanowire (CATSAI)	
	Pred. Neg.	Pred. Pos.		Pred. Neg.	Pred. Pos.		Pred. Neg.	Pred. Pos.
Act. Neg.	1625	313	Act. Neg.	1862	211	Act. Neg.	156	31
Act. Pos.	199	380	Act. Pos.	651	276	Act. Pos.	501	2312

Supplementary Table III. Bounds used for the 3D hypersurface plots.

Device	V_1 (V)	V_2 (V)	V_3 (V)	V_4 (V)	V_5 (V)	V_6 (V)	V_7
Si FinFET, origin	-6.5	-1.5	-1.5	-5.0	-	-	-
Si FinFET, limit	-2.5	0.0	0.0	-5.0	-	-	-
GeSi Nanowire, origin	0.0	0.56	0.0	1.1	0.0	-	-
GeSi Nanowire, limit	4.0	0.56	2.5	1.1	4.0	-	-
Ge/SiGe Heterostructure, origin	0.48	0.0	0.74	0.0	0.79	0.0	0.41
Ge/SiGe Heterostructure, limit	0.48	2.0	0.74	2.0	0.79	2.0	0.41

Supplementary Table IV. Gate voltage space explored by CATSAI and Random Search algorithms for each of the devices considered.

Device	V_1 (V)	V_2 (V)	V_3 (V)	V_4 (V)	V_5 (V)	V_6 (V)	V_7 (V)
Si FinFET, origin	-6.5	-1.5	-1.5	-6.5	-	-	-
Si FinFET, limit	0.0	0.0	0.0	0.0	-	-	-
GeSi Nanowire, origin	0.0	0.0	0.0	0.0	0.0	-	-
GeSi Nanowire, limit	4.0	2.5	2.5	4.0	4.0	-	-
Ge/SiGe Heterostructure, origin	0.0	0.0	0.0	0.0	0.0	0.0	0.0
Ge/SiGe Heterostructure, limit	2.0	2.0	2.0	2.0	2.0	2.0	2.0

Supplementary Table V. Differences in the configuration of the algorithm for each of the devices considered.

Device	V_{bias} (mV)	Noise Threshold (pA)	Segmentation Threshold (pA)	diag_trace: size (mV)	2d_lowres: size (mV)	2d_highres: size (mV)
Si FinFET	7.6	2	20	100	80 × 80	120 × 120
GeSi Nanowire	4	2	1000	200	150 × 150	200 × 200
Ge/SiGe Heterostructure	0.5	10	30	100	80 × 80	120 × 120

Supplementary Table VI. Total number of current maps labelled as positive (i.e. corresponding to the double quantum dot regime) found by each labeller (Labeller 1, 2, 3, 4) for each device and for each run of CATSAI. Runs marked with an asterisk were excluded because the cryostat temperature was slightly higher than base temperature.

Experiment	Iterations	Time (hours)	Labeller 1	Labeller 2	Labeller 3	Labeller 4
Si FinFET, run 1	250	3.47	2	2	2	3
Si FinFET, run 2	250	4.17	12	12	10	10
Si FinFET, run 3	250	3.62	5	5	5	5
Si FinFET, run 4	250	4.15	9	6	6	7
Si FinFET, run 5	250	3.30	9	9	6	8
Si FinFET, run 6	250	3.90	9	9	7	9
Si FinFET, run 7	250	3.30	3	2	1	3
Si FinFET, run 8	250	3.86	13	13	7	13
Si FinFET, run 9	250	3.25	4	4	4	4
Si FinFET, run 10	250	3.81	10	11	8	11
Si FinFET, run 11	250	3.57	5	5	5	6
Si FinFET, run 12	250	3.83	13	13	13	13
GeSi Nanowire, run 1	250	8.42	45	58	74	48
GeSi Nanowire, run 2	250	8.26	46	61	80	54
GeSi Nanowire, run 3	250	8.57	38	60	77	49
GeSi Nanowire, run 4	250	9.18	40	64	79	46
GeSi Nanowire, run 5	250	8.21	38	52	73	47
GeSi Nanowire, run 6	250	8.90	38	64	78	54
GeSi Nanowire, run 7	250	8.12	39	46	70	46
GeSi Nanowire, run 8	250	8.68	46	59	79	48
GeSi Nanowire, run 9	250	9.05	50	67	84	48
GeSi Nanowire, run 10	250	9.31	51	64	78	52
GeSi Nanowire, run 11	250	9.38	50	64	82	54
GeSi Nanowire, run 12	250	9.02	43	63	78	55
Ge/SiGe Heterostructure, run 1	250	3.38	2	4	5	3
Ge/SiGe Heterostructure, run 2	250	2.50	2	3	2	2
Ge/SiGe Heterostructure, run 3	250	2.39	1	1	0	1
Ge/SiGe Heterostructure, run 4*	250	3.17	1	2	0	1
Ge/SiGe Heterostructure, run 5	250	3.04	3	2	2	1
Ge/SiGe Heterostructure, run 6	250	3.66	2	3	4	3
Ge/SiGe Heterostructure, run 7	250	3.19	1	1	1	2
Ge/SiGe Heterostructure, run 8	250	2.81	2	1	2	1
Ge/SiGe Heterostructure, run 9	250	3.19	1	1	1	1
Ge/SiGe Heterostructure, run 10	250	3.22	1	0	1	1
Ge/SiGe Heterostructure, run 11	250	2.91	3	4	1	2
Ge/SiGe Heterostructure, run 12	250	3.50	1	2	2	1
Ge/SiGe Heterostructure, run 13*	250	3.42	2	2	2	3
Ge/SiGe Heterostructure, run 14*	250	3.31	4	3	5	3
Ge/SiGe Heterostructure, run 15	250	2.99	3	4	4	4

Supplementary Table VII. Total number of current maps labelled as positive (i.e. corresponding to the double quantum dot regime) found by each labeller (Labeller 1, 2, 3, 4) for each device and for each run of Random Search. Runs marked with a an asterisk were excluded because the cryostat temperature was slightly higher than base temperature.

Experiment	Iterations	Time (hours)	Labeller 1	Labeller 2	Labeller 3	Labeller 4
Si FinFET, run 1	250	1.62	0	0	0	0
Si FinFET, run 2	250	1.68	0	0	0	0
Si FinFET, run 3	250	1.69	0	0	0	0
Si FinFET, run 4	250	1.58	0	0	0	0
Si FinFET, run 5	250	1.64	0	0	0	0
Si FinFET, run 6	250	1.62	0	0	0	0
Si FinFET, run 7	250	1.51	0	0	0	0
Si FinFET, run 8	250	1.45	0	0	0	0
Si FinFET, run 9	250	1.49	0	0	0	0
Si FinFET, run 10	250	1.52	0	0	0	0
Si FinFET, run 11	250	1.63	0	0	0	0
Si FinFET, run 12	250	1.56	0	0	0	0
GeSi Nanowire, run 1	250	4.40	11	18	23	15
GeSi Nanowire, run 2	250	4.06	5	13	20	10
GeSi Nanowire, run 3	250	4.44	9	17	28	11
GeSi Nanowire, run 4	250	3.82	3	12	21	8
GeSi Nanowire, run 5	250	4.66	12	20	30	14
GeSi Nanowire, run 6	250	4.58	10	22	32	17
GeSi Nanowire, run 7	250	4.17	11	11	22	13
GeSi Nanowire, run 8	250	3.92	7	14	21	10
GeSi Nanowire, run 9	250	4.53	14	23	30	17
GeSi Nanowire, run 10	250	4.37	12	19	23	16
GeSi Nanowire, run 11	250	4.59	11	20	30	14
GeSi Nanowire, run 12	250	4.21	19	23	28	18
Ge/SiGe Heterostructure, run 1	250	2.22	1	1	1	1
Ge/SiGe Heterostructure, run 2	250	1.83	0	0	0	0
Ge/SiGe Heterostructure, run 3	250	1.82	0	0	0	0
Ge/SiGe Heterostructure, run 4	250	1.85	0	0	0	0
Ge/SiGe Heterostructure, run 5	250	1.89	0	1	0	0
Ge/SiGe Heterostructure, run 6	250	1.82	0	0	0	0
Ge/SiGe Heterostructure, run 7	250	1.72	0	0	0	0
Ge/SiGe Heterostructure, run 8	250	1.68	0	0	0	0
Ge/SiGe Heterostructure, run 9	250	1.69	1	2	1	1
Ge/SiGe Heterostructure, run 10	250	1.81	0	0	0	0
Ge/SiGe Heterostructure, run 11	250	1.95	0	0	0	0
Ge/SiGe Heterostructure, run 12	250	1.52	1	1	1	1
Ge/SiGe Heterostructure, run 13*	250	1.64	0	0	1	0
